# Accuracy of parents’ subjective assessment of paediatric fever with thermometer measured fever in a primary care setting

**DOI:** 10.1186/s12875-022-01638-6

**Published:** 2022-02-21

**Authors:** George Edwards, Susannah Fleming, Jan Y. Verbakel, Ann van den Bruel, Gail Hayward

**Affiliations:** 1 Nuffield Department of Primary Care Health Sciences, Radcliffe Observatory Quarter, Woodstock Road, Oxford, OX2 6GG UK; 2 Academic Centre for Primary Care, Department of Public Health and Primary Care, KU Leuven, Leuven, Belgium

## Abstract

**Background:**

Fever is a common symptom of benign childhood illness but a high fever may be a sign of a serious infection. Temperature is often used by parents to check for illness in their children, and the presence of a high temperature can act as a prompt to consult a healthcare professional. It would be helpful for GPs to understand how well parental assessment of the presence of fever correlates with temperature measurement in the clinic in order to incorporate the history of the child’s fever into their clinical assessment.

**Methods:**

Secondary analysis of a cross-sectional diagnostic method comparison study. Parents were asked whether they thought their child had fever before their temperature was measured by a researcher. Fever was defined as a temperature of 38 °C and higher using either an axillary or tympanic thermometer.

**Results:**

Of 399 children recruited, 119 (29.8%) were believed by their parents to be febrile at the time of questioning and 23 (6.3%) had a fever as measured by a researcher in the clinic. 23.5% of children with a parental assessment of fever were found to have a fever in the clinic. Less than 1% of children whose parents thought they did not have a fever were found to be febrile in the clinic. Having more than one child did not improve accuracy of parents assessing fever in their child.

**Conclusions:**

In the GP surgery setting, a child identified as afebrile by their parent is highly likely to be measured as such in the clinic. A child identified as febrile by their parent is less likely to be measured as febrile.

**Supplementary Information:**

The online version contains supplementary material available at 10.1186/s12875-022-01638-6.

## Introduction

Fever is a common symptom in benign childhood illnesses [1]. It is often used by parents to confirm illness in a child [2]. Fevers are a source of concern and worry for parents [3]. As a sign of more severe illness, which in rare cases may lead to brain damage and death [4, 5], fever in a child acts as a prompt for a GP visit [6–8]. From a GP perspective, parents presenting with febrile children are common. In out-of-hours care almost a third of contacts for children under the age of 12 are fever related [9, 10] whilst fever accounts for 20% of all visits to the paediatric emergency department [11]. Current clinical guidelines also use fever thresholds as part of algorithms for admission decisions [7] meaning temperature levels affect GPs’ management decisions. Single assessment of a child’s temperature in a GP surgery may not be the most informative way to measure fever, particularly given the poor agreement between different thermometer types [12] and prior administration of antipyretics by parents.

Parents of children presenting to primary care are likely to have assessed their child’s fever [13] and it is useful for GPs to understand the previous duration and trajectory of a child’s fever. It would be helpful to know how well parental assessment of the presence of fever correlates with temperature measurement in the clinic in order to incorporate the history of the fever into a holistic assessment of the child.

This study is a secondary analysis of a larger methods comparison study which found wide limits of agreement between infrared, axillary and tympanic thermometry [12]. In the method comparison study we concluded that peripheral temperature measurement should be interpreted cautiously and within the context of a holistic assessment of the child. In this secondary analysis, we aim to establish whether parental assessment of fever correlated with temperature measurement in a primary care setting.

## Methods

We used data collected during a prospective cross-sectional diagnostic method comparison study comparing two non-contact infrared thermometers with electronic axillary and tympanic thermometers [12]. Children between 0 and 5 years with any acute illness of a maximum of 14 days presenting to general practice (9 sites) or an out-of-hours (OOH) general practice service (1 site) in Oxfordshire (UK) were eligible for inclusion. Demographic information (age, gender, ethnicity, number of siblings) and history of the illness (e.g. duration) were recorded by a research assistant before the child’s temperature was measured using four thermometers. Parents were asked whether they thought their child had “fever now” (yes/no). If they answered in the affirmative, they were asked whether the child was “warm to touch”, whether they had “measured temperature at home”, or “other”. Parents were also asked whether their child had received fever medication in the previous 6 h, and if so what medication had been administered.

The sample size for the main study was calculated at 533 to give a 0.75 °C accuracy for each limit of agreement. This was reduced to 400 to give a 0.10 °C accuracy.

We calculated sensitivity, specificity, and positive and negative predictive values using a 2 × 2 table against a threshold of 38 °C using either an electronic axillary or tympanic thermometer. Since there is little evidence of agreement between axillary and tympanic thermometry [12], and both have similar agreement with rectal temperatures [14, 15], we used a combined reference standard for this study. We did a further sub-group analysis of the sensitivity, specificity, and positive and negative predictive values of parents with only one child and those with more than one child. We used R (3.5.1) for our analyses.

## Results

### Descriptive

We recruited 401 children between April 2017 and August 2018 and we obtained an axillary or a tympanic thermometer reading for 399 children with a median age of 19.8 months (IQR 9.5–40.6); 202 (50.6%) were boys (see Table 1 for an age group distribution) and 234 (58.6%) children had siblings. We were unable to obtain a temperature reading with either axillary or tympanic thermometers from two children. Using a threshold of 38.0 °C with either the axillary or tympanic thermometer 30/399 (7.5%) of children were febrile.

**Table 1:** Age distribution of 399 children with an axillary or tympanic temperature reading

Age group	*N*	%
< 1 month	5	1.3%
≥1 month and < 3 months	20	5.0%
≥3 months and < 12 months	100	25.1%
Between 12 months and 5 years	274	68.7%

The parents of 119/399 (29.8%) children thought their child had a fever at the time of asking. Of these, 67/119 (56.3%%) indicated that their child felt ‘warm to touch’, 41/119 (34.5%) had measured the temperature of their child at home, 10/119 (8.4%) used both techniques, and 1/119 (0.8%) did not specify. The parents of 134/399 (33.6%) children gave an antipyretic in the 6 hours prior to professional assessment in the clinic. The parents of 75/134 (56.0%) of these children receiving an antipyretic thought they had a fever.

### Methods comparison

Against a reference standard of 38 °C using either an axillary or tympanic thermometer, parents’ subjective assessment of fever was associated a sensitivity of 93% (95% CI 73–99%), a specificity of 75% (95% CI 71–80%), a positive predictive value of 24% (95% CI 16–32%) and a negative predictive value of 99% (95% CI 97–100%) The positive likelihood ratio was 3.78 (95% CI 3.09–4.63), and the negative likelihood ratio was 0.09 (95% CI 0.02–0.34). See Table 2 and Supplementary Table 1 for further details.

**Table 2:** Test characteristics of parents’ subjective assessment of fever

Index Test	Reference	*N*=	TP	FP	FN	TN
Parents’ subjective assessment	38oC using either the axillary or tympanic thermometer	399	28	91	2	278

We analysed the subgroups of 165 parents with one child versus 234 with more than one child (Table 3, full data in Supplementary Table 2). The sensitivity, specificity, and NPV in each group was similar. The PPV of parents with one child was 31% (95% CI 19–46) in comparison to 18% (95% CI 10–29) for parents with more than one child.

**Table 3:** Test characteristics of parents’ subjective assessment of fever by family size

Sub-Group	Index Test	Reference	*n*=	TP	FP	FN	TN
Parents with one child	Parental subjective assessment	38oC using either the axillary or tympanic thermometer	165	15	33	2	115
Parents with more than one child	Parental subjective assessment	38oC using either the axillary or tympanic thermometer	234	13	58	0	163

## Discussion

### Summary of results

Of 399 children recruited to our study with axillary or tympanic thermometer measurements 119 (29.8%) were believed to be febrile by their parents. Only 30 (7.5%) children were found to have a temperature of 38 °C or more measured using either the axillary thermometer or tympanic thermometer.

Compared with this reference standard, 93% children found to have a fever in the clinic had been assessed as febrile by their parents (sensitivity). Of children assessed by their parents as having a fever, 24% were found to have a fever in the clinic (PPV) compared with only 1% of children whose parents through they were afebrile.

We examined whether parental experience, which we assumed to be greater in those with more than one child, would result in better diagnostic accuracy. The sensitivity, specificity, and NPV was similar in parents with one child and parents with more than one child. The PPV of parents with one child was 31% in comparison to 18% for parents with more than one child, although there were overlapping confidence intervals.

### Comparison with the literature

A meta-analysis of tactile assessment of fever by caregivers found a combined sensitivity of 87.5%, specificity of 54.6% [16], positive likelihood ratio of 1.93 (1.39–3.67), and a negative likelihood ratio of 0.23 (0.15–0.36). Neither predictive value was calculated. A subsequent study conducted in a hospital found palpation to be associated with a sensitivity of 86%, a specificity of 48%, an NPV of 66% and a PPV of 76% [17] although prevalence was not reported. Our results differ in the specificity and positive likelihood ratio, likely due to the setting and inclusion of children with non-febrile illness. Of the 11 studies included in the meta-analysis, four included patients specifically with fever as part of their complaint and none were conducted in primary care. In contrast we included all children presenting with acute illness, including those where fever might not be expected to be present by parents. This may have resulted in more parents correctly identifying a lack of fever. In support of this our study had more true negatives than the other studies.

One other study found parents’ subjective assessment of fever in children presenting to ED to be associated with a sensitivity of 74%, a specificity of 86%, a PPV of 52% and an NPV of 94% [13]. The prevalence of fever in this study was higher than ours at 17%. We found a similar sensitivity and specificity, but a lower PPV than in the ED, likely because we had a lower prevalence of fever.

### Clinical relevance

Our results help clinician’s incorporate a parents’ assessment of fever into a holistic assessment of the child. A parent’s subjective assessment of fever at presentation is a poor predictor of fever measured in primary care whilst a history of normal temperature in the current illness episode can however be seen as reasonably reliable by the GP.

### Limitations

Our study has several limitations. In the UK, NICE Guidelines recommend measurement of temperature in children presenting with fever symptoms using electronic axillary thermometers, or tympanic thermometers in children aged 4 weeks and older [7]. Rectal measurements are no longer recommended yet both the axillary thermometer and the infrared tympanic thermometer are associated with wide limits of agreement with rectal temperature measurement [14, 15]. Although comparison with axillary or tympanic thermometry is the most relevant comparison to current practice in the UK, use of these imperfect reference standards may limit international comparison.

Our study may also be underpowered to assess the usefulness of parent’s subjective assessment of fever to GPs as it uses secondary data from a study with a different purpose [12] and we found a low number of children with a fever.

As we recruited before their initial assessment, we did not collect information on the underlying chief complain. Alongside the low prevalence found in our study for the presence of fever, this could limit the generalisability of our finds to high prevalence settings or to children with a different spectrum of presenting complaints.

### Further research

Further research into how parents identify a fever in a home setting and how temperature readings are interpreted in the context of their child’s illness will improve our understanding of actions performed by parents preceding contact with the health services. This understanding will enable clinicians to make better use of parents’ assessment of their child’s illness.

Further research is also needed into trajectories of body temperature over an illness episode in a child. This will help both parents and clinicians to contextualise and interpret a child’s temperature in relation to the course of the illness.

## Conclusions

In the GP surgery setting, a child identified as afebrile by their parent is highly likely to be measured as such in the clinic. A child identified as febrile by their parent is less likely to be measured as febrile.

## Supplementary Information

**Additional file 1: Supplementary Table 1.** Full analysis. **Supplementary Table 2.** Full analysis by number of children.

## Data Availability

All data analysed as part of this study is available in the manuscript.
