# Supplementary material for: Accuracy of parents’ subjective assessment of paediatric fever with thermometer measured fever in a primary care setting
Source: BMC Prim Care. 2022 Feb 21;23:30. doi: 10.1186/s12875-022-01638-6 (PMC8862558; doi:10.1186/s12875-022-01638-6)
Supplement: Supplementary file 1 — Additional file 1: Supplementary Table 1. Full analysis. Supplementary Table 2. Full analysis by number of children. [file 12875_2022_1638_MOESM1_ESM.docx]

| Index Test | Reference | N= | TP | FP | FN | TN | Febrile (95% CI) | Sensitivity (95% CI) | Specificity (95% CI) | PPV (95% CI) | NPV (95% CI) | LR+ (95% CI) | LR- (95% CI) |
| --- | --- | --- | --- | --- | --- | --- | --- | --- | --- | --- | --- | --- | --- |
| Parents’ subjective assessment | 38oC using either the axillary or tympanic thermometer | 399 | 28 | 91 | 2 | 278 | 8% (5-11) | 93% (78 -99 | 75% (71-80) | 24% (16-32) | 99% (97-100) | 3.78 (3.09-4.63) | 0.09 (0.02-0.34) |

**Supplementary Table 1. Full analysis**

| Sub-Group | Index Test | Reference | n= | TP | FP | FN | TN | Febrile (95% CI) | Sensitivity (95% CI) | Specificity (95% CI) | PPV (95% CI) | NPV (95% CI) | LR+ (95% CI) | LR- (95% CI) |
| --- | --- | --- | --- | --- | --- | --- | --- | --- | --- | --- | --- | --- | --- | --- |
| Parents with one child | Parental subjective assessment | 38oC using either the axillary or tympanic thermometer | 165 | 15 | 33 | 2 | 115 | 10% (6-16) | 88% (64-99) | 78% (70-84) | 31% (19-46) | 98% (94-100) | 3.96 (2.80-5.60) | 0.15 (0.04-0.56) |
| Parents with more than one child | Parental subjective assessment | 38oC using either the axillary or tympanic thermometer | 234 | 13 | 58 | 0 | 163 | 6% (3-9) | 100% (75-100) | 74% (67-79) | 18% (10-29) | 100% (98-100) | 3.81 (3.05-4.75) | 0 |

**Supplementary Table 2. Full analysis by number of children**
